# 2442 Listening for empathy: Audio narratives in DPT curriculum as a model for interprofessional education

**DOI:** 10.1017/cts.2018.217

**Published:** 2018-11-21

**Authors:** Jeffrey S. Farroni, Laura W. Farroni, Rebecca Russell

**Affiliations:** UTMB, Galveston, TX, USA

## Abstract

OBJECTIVES/SPECIFIC AIMS: (1) Evaluate the auditory narrative process as a learning experience for interviewer, editor, and interviewee. (2) Discuss methodologies for developing or selecting audio narratives and suggest how to effectively integrate them into the DPT curriculum, or thread into individual coursework. (3) Experience and appraise podcast components developed for a DPT psychosocial aspects of disability course. METHODS/STUDY POPULATION: Students were provided preassessment and postassessment on empathy. Other methodologies include conducting interviews, developing story boards, and editing audio narratives. RESULTS/ANTICIPATED RESULTS: Learner feedback indicated that course material was experienced in a way that deepens one’s understanding of the complex and challenging issues facing patient, caregivers, and themselves as they embark on their profession. DISCUSSION/SIGNIFICANCE OF IMPACT: The utility of integrating different modalities within coursework is to enrich learner experience to encourage self-reflection and awareness of not only their identity but that of multidisciplinary collaborators.

